# Expression and localization of the polarity protein CRB2 in adult mouse brain: a comparison with the CRB1^rd8^ mutant mouse model

**DOI:** 10.1038/s41598-018-30210-5

**Published:** 2018-08-03

**Authors:** Jorge F. Dolón, Antonio E. Paniagua, Vicente Valle, Alicia Segurado, Rosario Arévalo, Almudena Velasco, Concepción Lillo

**Affiliations:** 1 Institute of Neurosciences of Castilla y León, IBSAL, Cell Biology and Pathology, University of Salamanca, 37007 Salamanca, Spain; 2 Present Address: Department of Ophthalmology and Stein Eye Institute, University of California, Los Angeles, CA 90095 USA

## Abstract

Acquisition of cell polarization is essential for the performance of crucial functions, like a successful secretion and appropriate cell signaling in many tissues, and it depends on the correct functioning of polarity proteins, including the Crumbs complex. The CRB proteins, CRB1, CRB2 and CRB3, identified in mammals, are expressed in epithelial-derived tissues like brain, kidney and retina. CRB2 has a ubiquitous expression and has been detected in embryonic brain tissue; but currently there is no data regarding its localization in the adult brain. In our study, we characterized the presence of CRB2 in adult mice brain, where it is particularly enriched in cortex, hippocampus, hypothalamus and cerebellum. Double immunofluorescence analysis confirmed that CRB2 is a neuron-specific protein, present in both soma and projections where colocalizes with certain populations of exocytic and endocytic vesicles and with other members of the Crumbs complex. Finally, in the cortex of CRB1^rd8^ mutant mice that contain a mutation in the *Crb1* gene generating a truncated CRB1 protein, there is an abnormal increase in the expression levels of the CRB2 protein which suggests a possible compensatory mechanism for the malfunction of CRB1 in this mutant background.

## Introduction

Cell polarity is achieved through the coordinated functions of three evolutionarily conserved polarity complexes: Crumbs and Par (apical complexes) and Scribble (basal complex)^1–3^. The achievement of correct cell polarity is essential for carrying out different functions in many tissues, such as the accurate positioning of cell adhesions, secretion and cell signaling. In neurons, as in other epithelial-derived cells, the acquisition of polarity is particularly important during neuronal development^4^, especially for the establishment and maturation of the synaptic communications among neurons^4–6^. The CRB proteins are members of the Crumbs complex which is also comprised of proteins PALS1 and PATJ. The intracellular domain of CRB, the sole transmembrane member of the complex, establishes a scaffolding complex with other members of the Crumbs, Par and Scribble complexes, essential for the correct accomplishment of all these activities^3,7,8^. The formation of these specialized cell surface domains and the acquisition of cell polarity is highly dependent on the transport and docking of the polarity proteins towards their appropriate locations in the plasma membrane. The transport of the CRB proteins from the TGN (trans Golgi network) to the plasma membrane has been described to be mainly mediated by small GTPases such as some Rab members^9–11^. In neurons, the particular performance of the GTPases is crucial for the correct development of dendrites and axons^12^. Also, the turnover of these proteins are dependent on, not only the *de novo* exocytic pathway, but also on the recycling of components, mediated in many cases by the retromer protein complex, necessary to rescue them from proteolytic degradation. In fact, it has been described that VPS35, a central component of the retromer, is responsible for the proper recycling of the CRB proteins and their retrograde transport into endosomes, contributing to the stabilization of the levels of available CRB^9,13,14^.

So far, the CRB proteins have been found in many species ranging from invertebrates to mammals^15^. Three genes encode the Crumbs orthologues in humans and mice, *CRB1/Crb1*, *CRB2/Crb2* and *CRB3/Crb3*, all of them expressed not only in retina but also in other organs. Using RT-PCR and *in situ* hybridizations analysis, *CRB1/Crb1* was found in the brain, in hypothalamic regions and optic area, the tuberalis region of the hypothalamus, preoptic area and in the granular layer of the cerebellum, the hippocampal dentate gyrus, the olfactory bulb and the rostral migratory stream^16,17^. *CRB2/Crb2*, again through RT-PCR and *in situ* hybridization analysis, was found in kidney, retinal pigment epithelium and choroid, the adult brain and at low levels in the placenta, heart and lung^18,19^. Expression of the CRB2 protein has been shown in the retinal pigment epithelium^20^ and in the embryonic mouse brain^21^. Finally, *CRB3/Crb3*, the most ubiquitously expressed of the three members, has been described in many diverse epithelial-derived tissues, including the retina^22,23^. It is known that among the three, while all of them are transmembrane proteins, CRB1 and CRB2 share a similar molecular structure, since both have a long extracellular domain. CRB3 lacks of this domain and it has been shown to perform functions different to those carried out by CRB1 and CRB2^21,23–25^.

It has been shown that mutations in the Crumbs gene in *Drosophila* produce alterations in the organization of polarity in epithelial cells^26,27^. In mammals, CRB3 plays an important role in establishing epithelial polarity and during organogenesis^28^. Also, loss-of-function in CRB1 causes retinal dystrophies, including retinitis pigmentosa^17^ and Leber Congenital Amaurosis^29^ in humans, but interestingly, without showing any apparent brain dysfunction or alteration^17,29–32^. The CRB1^rd8^ mouse model has been employed to study CRB1 functions since it presents a single base mutation that generates a premature stop codon resulting in a truncated CRB1 protein that only contains the N-terminal extracellular domain, leading to retinal degeneration^33,34^. But, to our knowledge, there are no studies analyzing the brain phenotype of this mouse model.

A complete ablation of CRB1 and CRB2 proteins in mouse retina simulate human Leber congenital Amaurosis. Moreover, the lack of *Crb2* gene function causes abnormalities in the gastrulation process in mouse development, producing lethality at embryonic stage 12.5^19^. Also, it has been reported that mutations in the *Crb2* gene produce a phenotype resembling congenital nephrosis with cerebral ventriculomegalia^35^, suggesting this phenotype could be related with a ciliopathy^36^.

During neural development, neuroepithelial cells undergo several epithelial-mesenchymal transitions, implying particular changes in their structure and cell adhesion features so these cells acquire the capacity to migrate and settle in different areas of the brain^37^. This process requires modifications in the apico-basal polarity of polarized and joint cells, losing this polarization and junctions/connections with other cells to be able to migrate^38^. As mentioned above, the acquisition of cell polarity is a highly regulated process involving many different proteins, together with CRB2. Although CRB2 is necessary for embryonic development, acting as an essential regulator for neuronal differentiation during neurogenesis^39^, and its mutation is pleiotropic, causing both neurological and renal defects^35^, up to now no studies have explored the presence of CRB2 protein in the brain. Here we demonstrate that CRB2 is ubiquitously distributed throughout the adult mouse brain, although it is particularly present in the cortex and certain hypothalamic areas. In addition, we found that CRB2 is enriched in vesicular compartments, both in soma and in neuronal processes. In these vesicles, CRB2 seems to be travelling together with specific synaptic components in exocytic vesicles, and in some cases together with the small GTPase Rab8. Additionally, here we describe the presence of CRB2 together with the retromer that could be involved in the recycling of this protein from the plasma membrane via VPS35 association, at least in processes of mature neurons. Finally, we find that the expression of CRB2 is increased in certain cortex layers of the CRB1^rd8^ mutant mouse, which presents a defective CRB1 protein, suggesting a possible compensatory mechanism or a gain of function.

## Results

### Expression and distribution of CRB2 in mouse brain

**Figure 1:**
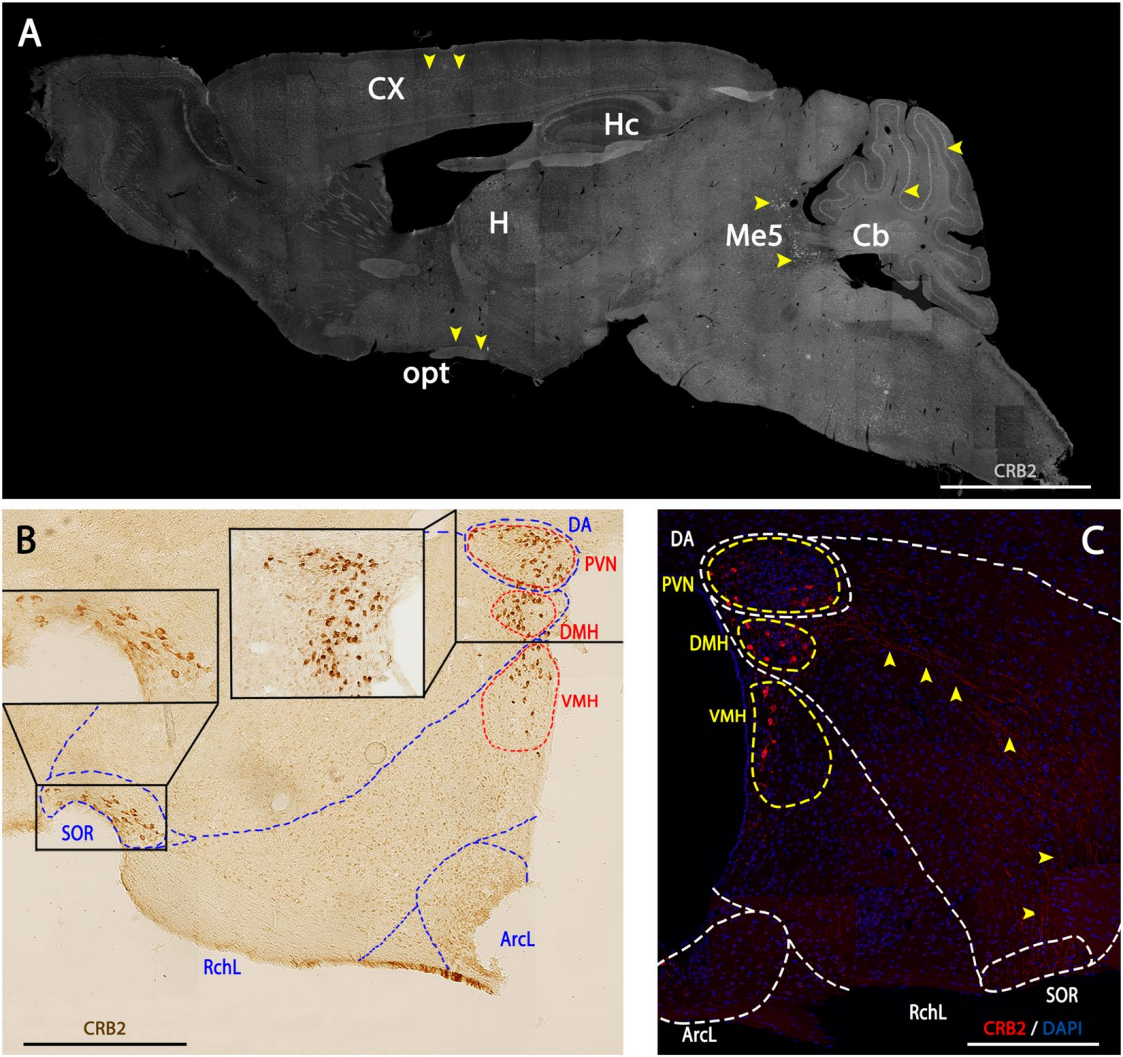
CRB2 localization in mouse brain. **(A)** Immunofluorescence of CRB2 in a sagittal section of the adult WT brain showing a punctate CRB2 labeling throughout the section, being more evident in certain nuclei of specific areas: Cortex (Cx), Hippocampus (Hc), cerebellum (Cb), Mesencephalic Trigeminal Nucleus (Me5), optic tract (opt) and Hypothalamus (H) (yellow arrowheads). (**B**) Immunohistochemistry of CRB2 in a coronal section of the hypothalamus. CRB2 labeling is especially evident in certain nuclei like the Dorsal area (DA), Periventricular Nucleus (PVN), Dorsomedial Hypothalamic Nucleus (DMH), Ventromedial Hypothalamic Nucleus (VMH), Lateral Retrochiasmatic area (RchL), Lateral Hypothalamic Arcuate Nucleus (ArcL), Retrochiasmatic Supraoptic Nucleus (SOR). **(C)** Immunofluorescence of CRB2 in a coronal adult mouse of the hypothalamic area showing a highly organized punctate labeling for CRB2 arranged from the DA to the SOR regions (yellow arrowheads). DAPI (in blue): nuclear labeling. Scale bars: 200 µm (**A**–**C**).

To determine the expression and spatial distribution of CRB2 in mouse brain, we performed immunofluorescence and immunohistochemistry analyses on sagittal and coronal mouse brain sections. The staining of CRB2 showed a punctate labeling with a broad and general distribution throughout the brain (Fig. 1A), although there were certain areas, such as the cortex (Cx), hippocampus (Hc), hypothalamus (H), the mesencephalic trigeminal nucleus (Me) and cerebellum (Cb), where the labeling was more evident, abundant and intense (yellow arrowheads). The immunohistochemistry analysis showed that in the hypothalamus, CRB2 was found concentrated in particular cell bodies, which were mainly located in specific areas such as the Periventricular Nucleus (PVN), Dorsomedial Hypothalamic Nucleus (DMH), Ventromedial Hypothalamic Nucleus (VMH) and the Retrochiasmatic part of the Supraoptic area (SOR) (Fig. 1B). Additionally, besides the nuclear staining, the immunofluorescence analysis showed a distinctive punctate CRB2 labeling that seemed to be accurately arranged between the Hypothalamic Dorsal Area (DA) and the SOR region (Fig. 1C, yellow arrowheads). Due to the particular enrichment of CRB2 labeled structures in the hypothalamus, the rest of the analyses carried out to discern the type of cells, processes and subcellular compartments containing CRB2, were performed in this particular area of the brain.

### In brain, CRB2 is exclusively present in neurons

**Figure 2:**
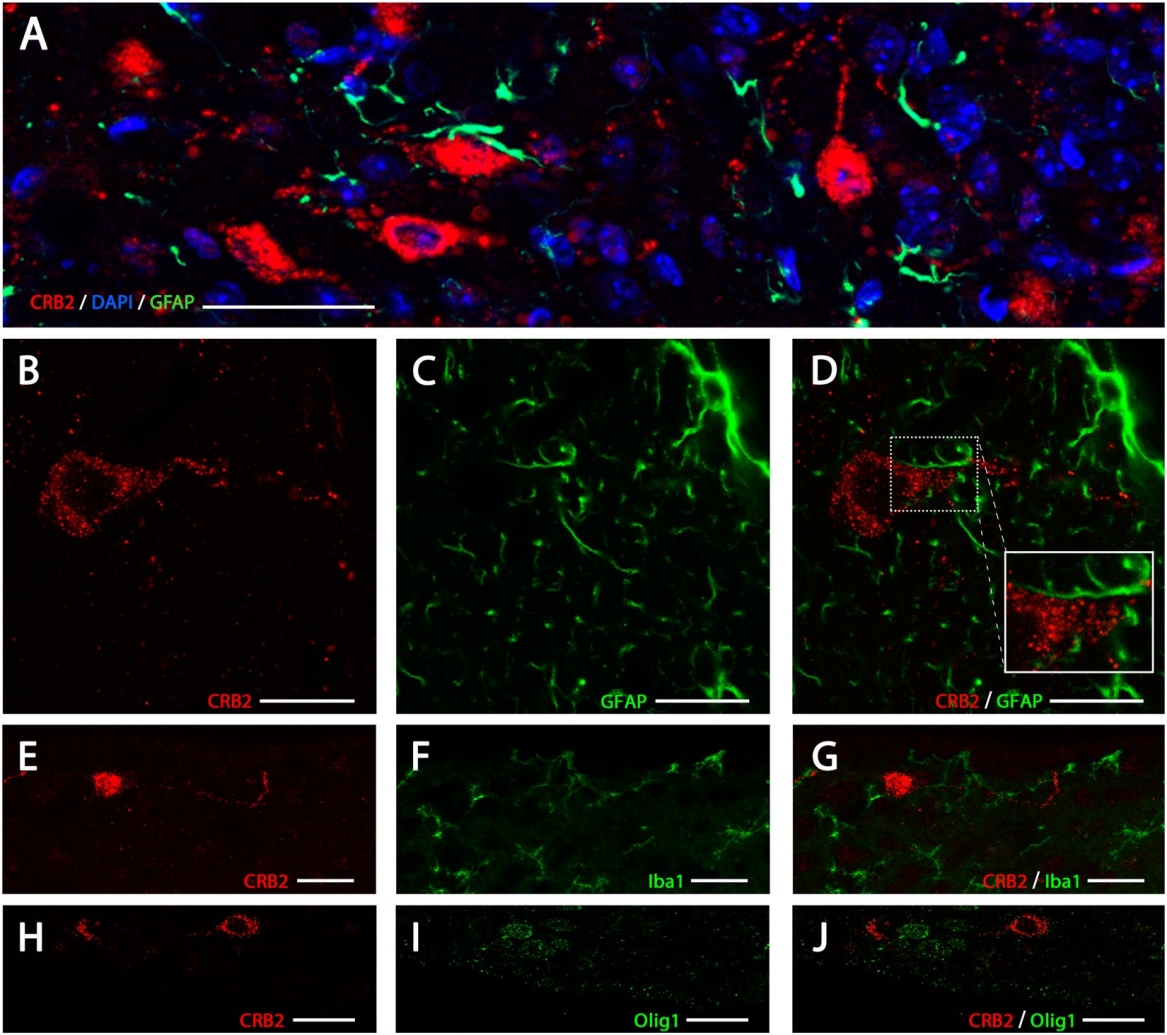
CRB2 immunofluorescence identification in the nervous system. Double immunofluorescence for CRB2 (**A**,**B**,**D**) and GFAP (**A**,**C**,**D**) in coronal sections of adult mouse PVN, showing that the two proteins do not colocalize in any of the cells or profiles. Double immunofluorescence labeling for CRB2 (**E**,**G**,**H**,**J**) and Iba1 (**F**,**G**) or Olig1 (**I**,**J**) in coronal sections of adult mouse PVN, showing that CRB2 is not present in microglia or oligodendrocytes. DAPI (in blue): Nuclear labeling. Scale bars: 100 µm (**A**), 15 µm (**B–D**), 20 µm (**E–J**).

To identify the cell types expressing CRB2 in mouse brain, double immunofluorescence analysis of CRB2 and proteins specific for certain cell types were performed, such as GFAP (Glial Fibrillary Acidic Protein) to identify astrocytes (Fig. 2A–D), Iba1 (Ionized calcium-biding adapter molecule 1) to identify microglia (Fig. 2E–G) or Olig1 (Oligodendrocyte Transcription Factor 1) for oligodendrocytes (Fig. 2H–J). CRB2 was present as punctate cytoplasmic staining in large round cells (Fig. 2) and in scattered profiles in a pattern resembling cell’s processes. This staining did not overlap with GFAP (Fig. 2A–D), since the GFAP+ profiles appeared to surround the CRB2+ labeled cell bodies and profiles, proving that CRB2 was not present in astrocytes. Similarly, CRB2 labeling did not colocalize with Iba1+ profiles (Fig. 2E–G) or oligodendrocyte+ labeling (Fig. 2H–J). The shape and size of the CRB2 labeled cell bodies and the absence of colocalization with glial markers proved that in mouse brain, CRB2 is only present in neurons.

**Figure 3:**
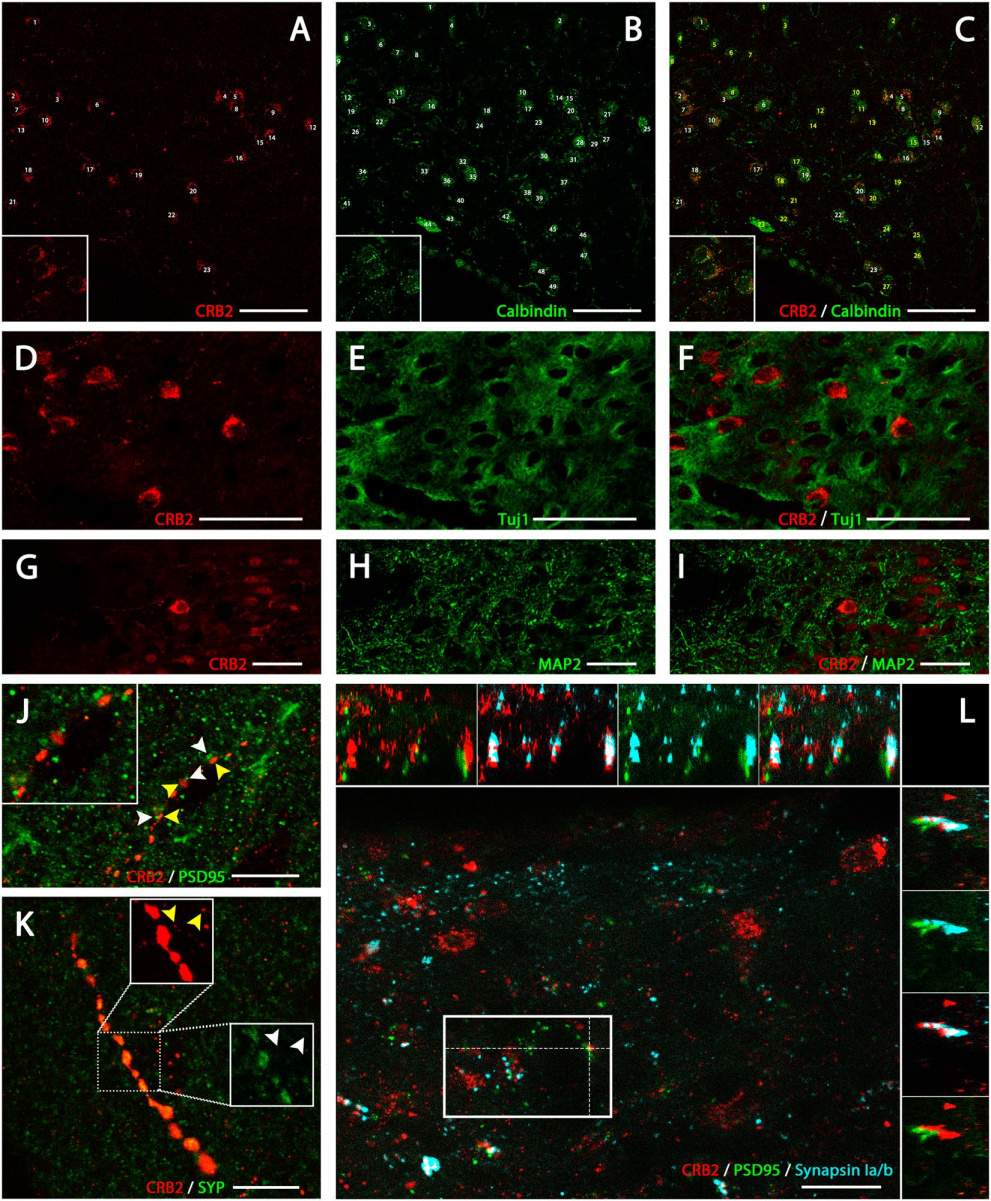
Immunofluorescence localization of CRB2 in cell bodies and profiles. Double immunofluorescence for CRB2 (**A**,**C**,**D**,**F**,**G**,**I**) and neuronal markers as Calbindin (**B**,**C**), Tuj1 (**D**,**F**) or MAP2 (**G**,**I**) in coronal sections of adult mouse PVN. Quantification of CRB2+ neurons (**A**,**C**) and Calbindin+ (**B**,**C**), showing that most of the Calbindin+ neurons are also CRB2+, and that all CRB2+ neurons, are Calbindin+ (white numbers in A and C). Double immunofluorescence for CRB2 (**D**,**F**,**G**,**I**) and Tuj1 (**E**,**F**) or MAP2 (**H**,**I**), neuronal cytoskeletal proteins, showing that CRB2+ is present in the cell bodies surrounded by the Tuj1+ and MAP2+ neuronal projections. Double immunofluorescence for CRB2 (**J–K**) and PSD95 (**J**) or SYP (**K**), in coronal sections of adult mouse PVN. (**J**) CRB2 (yellow arrowheads) and PSD95 (white arrowheads) do not colocalize in the punctate profiles, but some spots are opposite one another. (**K**) CRB2/SYP colocalization is frequent in the profiles scattered throughout the sections, but there are some SYP profiles (white arrowheads) which are nor labeled for CRB2 and vice versa (yellow arrowheads). (**L**) Projection along the z-axis [Plan-Apochromat 63x/1.40 Oil DIC M27 objective; Z-stack: 109 slices (25,92 μm)] of a triple immunofluorescence for CRB2 (red), PSD95 (green) and synapsin Ia/b (light blue) in a coronal section of adult mouse PVN. Orthogonal views (XY images above and YZ on the right side) showed that CRB2 and PSD95 not only do not colocalize but also are opposite one another, CRB2/synapsin Ia/b colocalization is frequent and the presynaptic CRB2/Sinapsin Ia/b+ profiles are facing the PSD95+ postsynaptic profiles. Scale bars: 50 µm (**A**–**I**), 10 µm (**J**,**K**), 20 µm (**L**).

**Figure 4:**
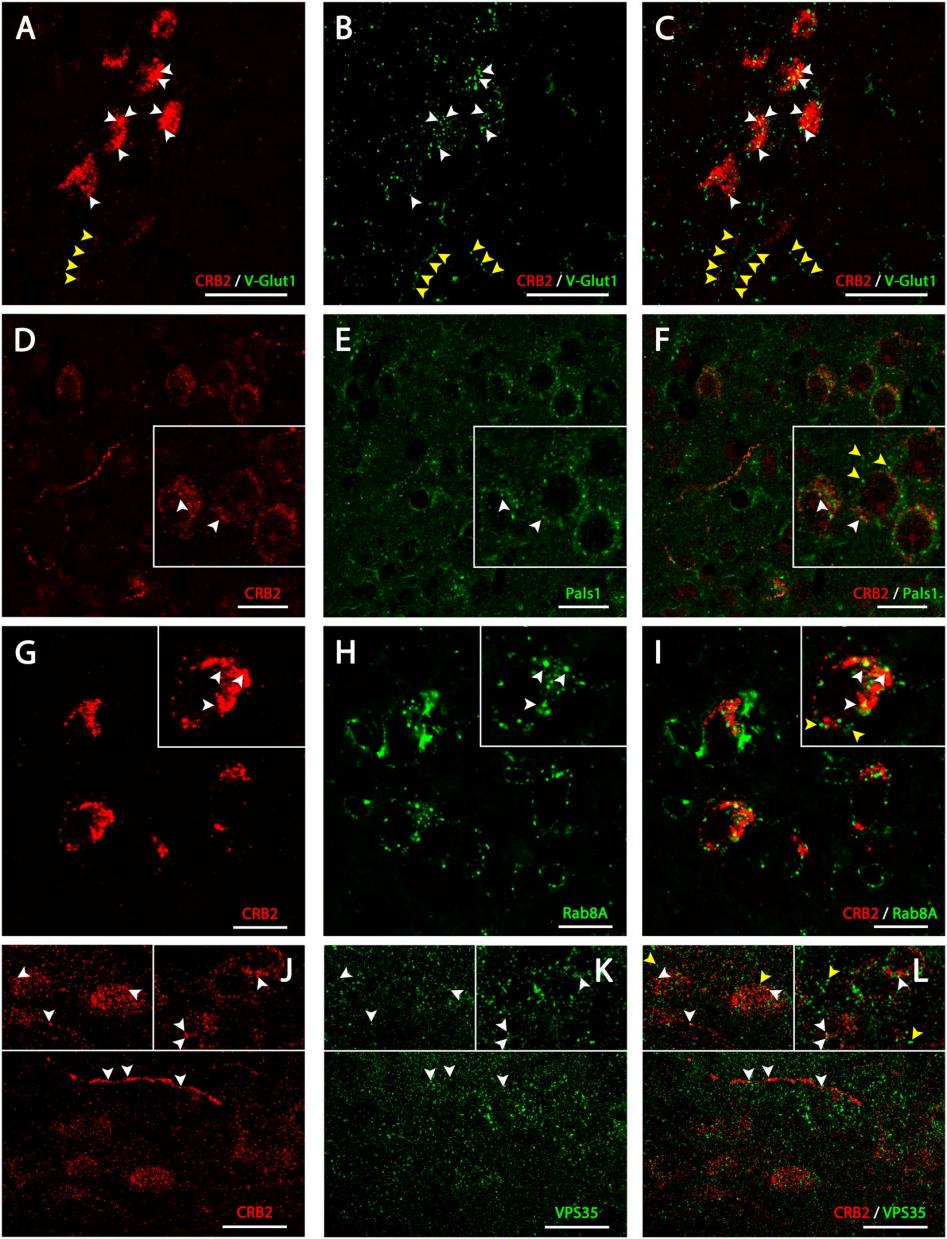
CRB2 localization in vesicular compartments in the cell bodies of neurons. Double immunofluorescence of CRB2 (**A**,**C**) and V-Glut1 (**B**,**C**) and CRB2 (**D**,**F**) and Pals1 (**E**,**F**) in coronal sections of adult mouse PVN. (**A**–**C**) The CRB2 punctate labeling found in the cell bodies partially colocalize with some V-Glut1 labeled profiles (white arrowheads). (**D–F**) CRB2 (arrowheads in D) and Pals1 (arrowheads in E) frequently colocalize in the labeled profiles in both soma and in cell processes, but there are some Pals1 profiles which are not labeled for CRB2 and vice versa (yellow arrowheads). Double immunofluorescence analysis of CRB2 (**G–I**) and Rab8A (**H–I**) and CRB2 (**J–L**) and VPS35 (**K–L**) in coronal sections of adult mouse PVN. CRB2 labeling (arrowheads in G) in cell bodies colocalize partially with Rab8A (arrowheads in H and I), but there are some Rab8A profiles independent of CRB2 (yellow arrowheads in I). CRB2/VPS35 labeling shows partial colocalization throughout sections, but there are VPS35 positive profiles not labeled for CRB2. Scale bars: 20 µm (**A–C**), 100 µm (**D–F**), 10 µm (**G–I**), 20 µm (**J–L**).

This inference was confirmed by performing double immunofluorescence experiments for CRB2 and typical neuronal cells markers, such as the calcium binding protein calbindin (Fig. 3A–C), Tuj1 (neuron specific class III beta tubulin) (Fig. 3D–F) or MAP2 (microtubule associated protein 2) (Fig. 3G–I). Calbindin is expressed by the vast majority of the neurons in the PVN of the hypothalamus^40^, the region where CRB2 was abundantly present in cell bodies, being then chosen to characterize its subcellular expression. The double immunofluorescence labeling with the neuronal marker for calbindin (Fig. 3A–C), showed that CRB2 is expressed in a subset of calbindin+ neurons and in fact, all CRB2+ cell bodies were also calbindin+ (shown with white numbers in Fig. 3A,C), but not the opposite (yellow numbers in Fig. 3C). Double immunofluorescence for CRB2 and neuronal cytoskeletal markers, such as Tuj1 (Fig. 3E,F) or MAP2 (Fig. 3H,I) shown that CBR2 (Fig. 3D–I) was present in the soma and in the projections of neurons identified with these markers. To try to identify the structures with a punctate pattern labeled for CRB2 resembling cell processes, we performed double and triple immunofluorescence experiments for CRB2 and proteins present in specific cell compartments, such as synaptic terminals (Fig. 3J–L) and diverse types of vesicles (Fig. 4). Different antibodies were used to specifically distinguish postsynaptic PSD95 profiles (Post Synaptic Density 95) (green in Fig. 3J,L) from presynaptic terminals, labeled for the protein synaptophysin (SYP) (Fig. 3K) or synapsin Ia/b (light blue in Fig. 3L), specifically present in presynaptic vesicles. This determined that PSD95 did not colocalize with the CRB2+ profiles on cellular processes, as shown in Fig. 3J and in the orthogonal views shown in the upper and right margins of Fig. 3L; or within the punctate labeling found in cell bodies (Supplementary Fig. 1A–C). Also, The CRB2/SYP double immunofluorescence analysis showed that these two proteins rarely colocalized in the cell bodies (Supplementary Fig. 1D–F), but frequently colocalized in the same punctate profiles resembling cells’ processes (Fig. 3K). Also, there were CRB2+ and SYP+ profiles which did not colocalize (arrowheads in Fig. 3K). Interestingly, some CRB2+ profiles were clearly opposite to the particles labeled for PSD95, as shown by arrowheads in Fig. 3J, that the triple immunolabeling for CRB2/synapsin Ia/b/PSD95 demonstrated to be presynaptic terminals labeled for both CRB2 and synapsin Ia/b (white labeling, as the result of the cololocalization of red CRB2 and light blue synapsin Ia/b, in the upper and right-sided orthogonal views shown in Fig. 3L) facing PSD95+ postsynaptic profiles (green in Fig. 3L). Then, this triple immunofluorescence analysis proved that CRB2 is present in some presynaptic terminals which are usually facing the PSD95+ postsynaptic elements and that CRB2 is not expressed in post-synaptic profiles (demonstrated by the lack of colocalization in the orthogonal views of Fig. 3L).

Double immunofluorescence analysis for CRB2 and V-Glut1, the vesicular glutamate transporter expressed in excitatory synaptic vesicles (Fig. 4A–C), showed some colocalization of these two proteins in the soma of neurons of the PVN (white arrowheads in Fig. 4A–C), although not all profiles labeled with V-Glut1 in the somas were CRB2+ and vice versa. Also, the CRB2 labeling found in the projections resembling cells’ processes did not colocalize with the V-Glut 1 labeling (yellow arrowheads in Fig. 4A–C). Since CRB2 is a member of the Crumbs polarity protein complex, we analyzed whether it was co-expressed with another Crumbs complex member, Pals1. The CRB2/Pals1 double immunofluorescence analysis showed that there was partial colocalization of these two proteins in the same group of neurons (arrowheads in Fig. 4F), but there was also Pals1 labeling independent of the CRB2+ profiles (yellow arrowheads in Fig. 4F).

Finally, since CRB2 is a transmembrane protein, it should be specifically transported to and from the plasma membrane throughout its lifespan via vesicular trafficking, similar to other transmembrane proteins. To address this, a double immunolabeling for CRB2 and proteins specifically found in vesicles related with the exocytic and endocytic vesicle trafficking pathway was performed. The CRB2/Rab8A (Fig. 4G–I) and CRB2/VPS35 (Fig. 4J–L) immunofluorescence analysis showed that CRB2 a partial colocalization with VPS35, a component of the retromer complex (white arrowheads in Fig. 4J–L), both in soma and projections, although not all VPS35+ profiles where CBR2+ (yellow arrowheads in Fig. 4J–L) and vice versa. Therefore, CRB2 is present at least, in some vesicles trafficking from the cell membrane through a retromer-controlled endocytic pathway. The CRB2/Rab8A immunofluorescence analysis showed partial colocalization (white arrowheads in Fig. 4I), suggesting that in some vesicles, CRB2 is travelling together with Rab8A through the same exocytic pathway, although not all Rab8A vesicles contained CRB2 and vice versa (yellow arrowheads in Fig. 4I).

### Differential expression of CRB2 in WT and CRB1^rd8^ adult mouse brain

**Figure 5:**
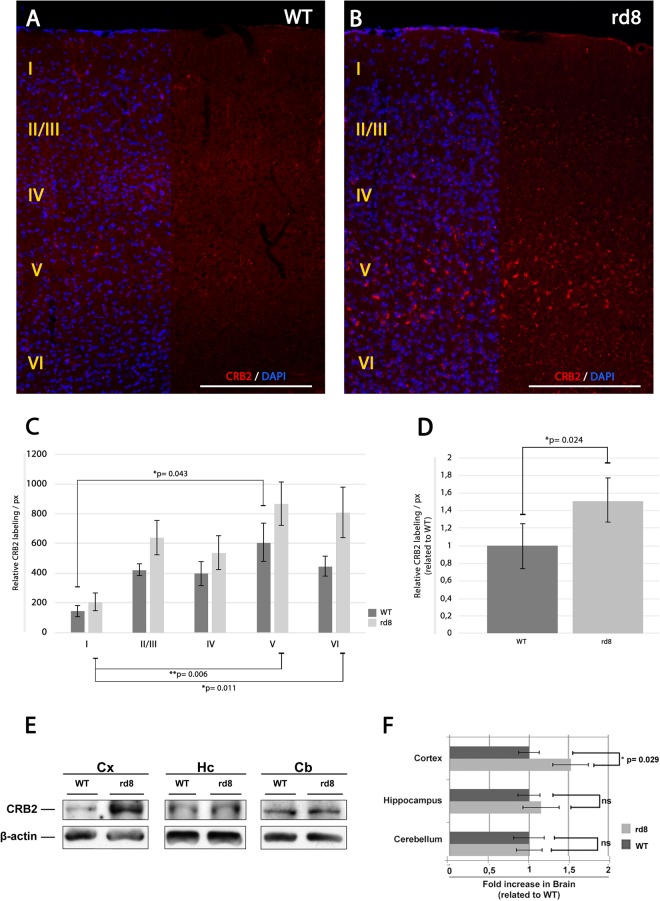
CRB2 distribution in the mouse cortex. (**A–B**) Immunofluorescence CRB2 labeling of a sagittal section of the adult WT (**A**) and CRB1^rd8^. (**B**) Cortex of the mouse brain showing the protein distribution throughout the layers of the cortex. (**C)** Graph showing the quantification of the CRB2 relative labeling/px in the different layers of the cortex of WT and CRB1^rd8^ mice. (**D**) Graph showing the normalized quantification of the CRB2 relative labeling/px in the whole CRB1^rd8^ mouse cortex related to WT. (**E–F**) WB analysis of the expression of CRB2 in adult WT and CRB1^rd8^ mice brain. (**E**) Comparative CRB2 (150 kD) protein expression in WT and CRB1^rd8^ mice cortex, hippocampus and cerebellum areas. Blots were cropped from the same gel and quantitative comparisons were performed between samples of the same blot. Full-length blots are presented in Supplementary Fig. 4. (**F**) Graph obtained from data in E showing the comparative quantification of CRB2 protein expression in WT and CRB1^rd8^ mice cortex, hypothalamus and cerebellum. β-actin (42 kD) was used as the loading control. DAPI (in blue): nuclear labeling. Scale bars: 200 µm. Data are presented as mean ± s.e.m. Statistical information: The Kolmogorov Smirnov test was used to assess the normality of sample distribution. In **C-WT**, more than three groups were analyzed with one way-factorial ANOVA and the Bonferroni’s post-hoc test (n = 3). In **C-rd8**, more than three groups were analyzed with Kruskal Wallis test (n = 3). In **D**, two experimental groups were compared with the Student’s T test (n = 3). In **F**, in all cases, two experimental groups were compared with the Mann Whitney U test (n = 15). Asterisks indicate statistical differences. Two asterisks indicate highly statistical differences.

To try to discern whether the dysfunctional CRB1 protein in the CRB1^rd8^ mouse brain may cause any phenotype related with the expression of CRB2 in this organ, based on their similarity in structure and functions in other tissues, we compared the expression of CRB2 in WT and CRB1^rd8^ mice brains. CRB2 was found in a punctate distribution throughout diverse brain areas such as cortex (Figs 1A, 5A,B) hippocampus and cerebellum (Fig. 1A, Supplementary Fig. 2A–D). In the hippocampus, CRB2 protein expression was more abundant in the CA3 area (Supplementary Fig. 2A,B) and in cerebellum, CRB2 protein expression was enriched in the Purkinje layer (Supplementary Fig. 2C,D). Comparing the CRB2 protein expression in hippocampus (Supplementary Fig. 2B), cerebellum (Supplementary Fig. 2D), hypothalamus (Fig. 1B,C, Supplementary Fig. 2E,F) and SOR area (Fig. 1B,C, Supplementary Fig. 2H) of WT and CRB1^rd8^ mouse models, no major differences were observed regarding the intensity, distribution or enrichment of the CRB2 labelling. On the other hand, the organization of the cortex into segregated layers allowed us to better analyze the precise distribution of the punctate staining of CRB2 in these layers. It was observed that CRB2 was not evenly distributed throughout the different cortical layers; being particularly enriched in layers II/III and V (Fig. 5A). Interestingly, we found that the expression of CRB2 in the cortex of the CRB1^rd8^ mouse was apparently more abundant than that of the WT (Fig. 5A,B). ImageJ software was employed to quantify the immunolabeling of CRB2 in three animals of each genotype (WT and CRB1^rd8^). First, we quantified the labeling of each separate layer and compared the differences in labeling among layers. To analyze the labeling within the different layers, the cortex was reconstructed using images from each section of every animal included in the study, and then the cortex layers were individually analyzed using the ImageJ software. Using the threshold tool, the labeling intensity of CRB2 was resolved over a black background and the pixel size and circularity of the smallest particle labeled was used as the minimum baseline for quantification. This analysis was performed in five different areas in each cortex layer of the animals analyzed to standardize the data. By using these parameters, we obtained a number of CRB2 positive particles for each cortex layer and genotype, and a relative CRB2 labeling/pixel. This configuration of size and circularity, was constant in all of the experiments and animals analyzed. This analysis demonstrated that in both phenotypes, most of the CRB2 protein is predominantly localized in layer V (Fig. 5C). Although this labeling intensity was not statistically different among the same layers of the two genotypes, statistically significant differences between layers I and V were detected in both WT and CRB1^rd8^ mice, and between layers I and VI, but only in CRB1^rd8^ mouse (Fig. 5C). The lower p-values in the mutant mouse indicated a greater difference in CRB2 labeling in this background. When analyzing the CRB2 labeling in the entire area of the cortex without distinguishing any of the layers, the statistical results showed that there was a significant difference in the amount of immunofluorescence in the cortex of the two genotypes analyzed (Fig. 5D).

The expression of CRB2 in the brain of CRB1^rd8^ and WT mice was also analyzed by WB. The differential WB analysis of the cortex (Cx), hippocampus (Hc) and cerebellum (Cb) areas (Fig. 5E) showed that at least in Cx there is a statistically significant increase in the amount of CRB2 protein in the mutant compared to WT (Fig. 5F). This data verifies the results obtained with the immunofluorescence analysis, where the signal of CRB2 was higher in the Cx of CRB1^rd8^ mouse mutant compared to control (Fig. 5A,B) and no differences in the intensity or enrichment of CRB2 in CRB1^rd8^ Hc and Cb areas were found compared to control (Supplementary Fig. 2). In order to elucidate whether the increase in CRB2 in CRB1^rd8^ brain, as shown by immunofluorescence and WB, could modify the expression of other proteins known to be directly associated with CRB, we performed WB analysis of some of them. Certain members of the Par complex, such as Par3, involved in the acquisition of cell polarization, and proteins involved in the establishment and maintenance of the cell-cell adhesions, such as β-Catenin and occludin, were analyzed (Supplementary Fig. 3A). Even though a relative increase in the level of β-catenin in the Cx and Cb was apparent (Supplementary Fig. 3B) we did not detect any statistically significant differences in the levels of expression of any of these proteins when comparing CRB1^rd8^ and WT tissue samples.

## Discussion

### CRB2 expression in brain

In this study, the expression of CRB2, one of the three members of the CRB protein family, in adult mouse brain, was investigated. Previous works have shown the expression of CRB1 in brain^16^, but to date there is no data regarding the expression and localization of CRB2 in this tissue; although it has been shown to be present in the mouse brain during embryogenesis^21,41^, in the retinal pigment epithelium^20^ and in neural retina^42,43^. Although previous reports have suggested the presence of CRB2 mRNA in neurons^39^, the lack of evidence supporting this could be due to the absence of proper tools that would have allowed more detailed studies. Thus, using a specific polyclonal antibody designed by our laboratory^20^ our work shows, for the first time, the expression of the CRB2 protein in adult mouse brain, where it is enriched in certain areas. Also, the detailed confocal microscopy images of the double and triple immunofluorescence analyses clearly showed that CRB2 is exclusively expressed in subsets of neurons, in certain vesicular structures distributed in their cell bodies and in some processes. The experiments performed in our study could not reveal why only specific neurons located in certain brain areas expressed a higher amount of CRB2 than others, or whether the CRB2 + neurons from different brain areas had something in common. Further and more detailed studies to find out the specific role that CRB2 may have in these neurons and the analogies that they share are needed to solve these questions.

### CRB2 subcellular localization

When analyzing the distribution of CRB2 in mouse brain, a condensed punctate labeling in the soma of some neurons was detected, as well as another labeling that resembled projections of these cells. Since CRB2 is a transmembrane protein and its final destination is the plasma membrane, it was expected that the punctate labeling observed in the soma was associated with vesicles, both those trafficking from the endoplasmic reticulum to the Golgi complex and those aimed to the plasma membrane as the protein is required to travel towards its target. This would also include those vesicles responsible for recycling the protein into endosomal compartments when endocytosis of the protein is required, as well as other transmembrane components. The retromer complex is a key component involved in recycling endocytosed proteins from endosomes or the plasma membrane to the trans-Golgi network^13,44^. The retrograde trafficking led by the retromer complex is crucial for the establishment of epithelial cell polarity, allowing the mediation of CRB to define the apical membrane^14,45^. Likewise, it has been shown to preserve brain homeostasis through its role in preserving the progress of neurodegeneration^46^. In other systems, retromer had already been implicated in the correct internalization and recycling of CRB proteins from the plasma membrane, sorting them away from the degradative route, with the purpose of ensuring their availability and efficiency for turnover and cell-membrane docking^9^. Our results show that most of the CRB2 protein in neurons is enclosed in vesicles, and confirm, as has been shown in other cell types, that the recycling of CRB2 from the cell membrane is accomplished, in part, by the retromer protein complex, revealed by the CRB2/VPS35 colocalization, being VPS35 a major component of the retromer complex^47^; and taking into account that at least in *Drosophila*, Vps35 interacts with Crb and regulates its levels^14,45^. Regarding the vesicles of the exocytic pathway, some of the synaptic vesicles containing V-Glut1, a vesicular glutamate transporter essential in the synaptic transmission, as it mediates the transport of glutamate into vesicles^48^, contained CRB2, but only in the soma. Thus, this protein would travel together with glutamate in some cases and also with the small GTPase Rab8A protein, since our results confirmed that CRB2 is partially carried through the exocytic pathway together with these proteins. Rab8-containing vesicles seem to be involved in the growth of neurites during the development of PC12 cells *in vitro*^49^, suggesting its role in this same process *in vivo*. Additionally, it has been previously shown that Rab8A mediates the transport of *de novo* generated CRB2-containing vesicles towards the plasma membrane^9–11^, supporting the idea that this transport is important for the stabilization of the protein levels of members of the Crumbs complex. Additionally, our results also showed that some neuronal presynaptic terminals contain CRB2 together with synaptophysin or synapsin, proteins specific to the pre-synaptic vesicles, and this colocalization seems to occur preferentially in the neuronal projections, not in the soma. Therefore, it is probable that one of their final targets may be the SYP+ or synapsin+ synaptic vesicles, once the CRB2+/SYP- vesicles observed in the soma have reached the synaptic terminals. Our study reinforces this idea, although further experiments need to be performed in order to provide evidence of this in our model, and other components involved in this exocytic pathway deserve further investigation in the future.

Also, our results show that CRB2 is especially abundant in calbindin+ neurons, a calcium binding protein with broad expression in neurons^50^ but mostly within the hypothalamic lateral area^51^. In fact, our data suggest that all neurons containing CRB2 in the soma were calbindin+, but not the opposite. All these results, together with the subcellular localization of CRB2, such as the presence of CRB2/Pals1 colocalization in these neurons expressing calbindin and its colocalization with some of the SYP+ or synapsin vesicles exclusively in the pre-synaptic terminals and V-Glut1+ profiles, seem to be in line with an hypothesis raised by other groups proposing that CRB2 may have a role in signaling and neuronal communication in the adult mouse brain. And that this role may be dependent on the final location of the protein and on the highly regulated exo- and endocytotic events. For example, a previous study has shown that a complete ablation of CRB1 and CRB2 proteins in mouse retina simulate a serious retinal disorder in human, and produces a dysregulation in the proliferative signaling pathway YAP/Hippo in the retinal progenitor cells^29^.This hypothesis is consistent with many independent studies demonstrating the association between CRB proteins and different cell signaling pathways related with cell proliferation, differentiation and cell-cell adhesion have been proposed and studied in different organisms. For instance, some studies have found an increased level of calcium activity during neuronal signaling and neuronal communication in calbindin-positive neurons^52–54^, suggesting the importance of the levels of this molecule for this process. Also, in *Drosophila*, the protein homologous to CRB in these organisms has been shown to repress Notch activity, probably by direct interaction and also by limiting gamma-secretase complex activity, one of the proteases involved in Notch processing^55^. Also, CRB proteins seem to mediate Hippo signaling, a pathway which directly regulates cell proliferation and apoptosis^56^. It has been also shown that in *Drosophila*, the homologous of Pals1 (a member of the Crumbs complex) that interacts with the cytoplasmic domain of CRB1^57^ and with CRB2^58^ plays an important role in the synaptic junctions by recruiting or releasing neurotransmitters to or from the synaptic vesicles^59^. It is also known that in mammalian brain, the expression of proteins of the MAGUK family, such as Pals1, occurs at synaptic junctions and have a central role regulating synaptogenesis, clustering synaptic receptors, organizing signal transduction flatways and modulating synaptic plasticity^60^. Finally, our results showing the localization of CRB2 in different layers of the cortex and the latest studies performed in embryos demonstrating that CRB2 cooperates with Pals1 for the correct development of the cortex^58^, are data that support the hypothesis that in adult mouse brain, CRB2 may lead to the correct establishment of the polarity of neurons and to the interaction with other proteins in the signal transduction pathway to facilitate the correct development of these areas.

### CRB2 protein expression is more abundant in CRB1^rd8^ mouse brain

The CRB1^rd8^ mouse has a deficit in the CRB1 function^33,34,61^, and our study shows that at least in cortex, there is an increase in the expression level of the CRB2 protein. It is known that CRB1 and CRB2 share a similar molecular structure and, interestingly, in the retina both are co-expressed in the same cell types; for example, in the membrane of Müller cells near the outer limiting membrane, where they could be performing similar functions^43^. In addition, although it has not yet been proven, it has been suggested that in some cell types these two proteins are redundant. For instance, some compensatory mechanisms may occur in retinal Müller glial cells, suggesting that the level of one of the proteins could become increased when the other protein becomes defective, although this theory has yet to be shown in this cell type^62^. Our data supports this idea, since defects in the function of CRB1 seem to generate an increase in CRB2 expression in the CRB1^rd8^ mouse brain, which could indicate a compensatory effect in response to this malfunction. This compensatory effect (or gain of function) could also explain the fact that no variation in the relative amounts of proteins such as Par3, β-Catenin and Occludin were observed, whose roles are associated with those of CRB proteins. However, we did notice an increasing trend in β-Catenin protein levels in the CRB1^rd8^ mouse brain areas analyzed. It is known that both CRB2 and β-catenin have crucial roles during gastrulation^62,63^ due, in part, to the importance of the correct function of polarity proteins during the differentiation of progenitor cells via signaling pathways such as Wnt^7,62,64^. Additionally, it has been described that the removal of CRB2 produces a decrease in the presence of adherens junction proteins, like β-catenin, during brain development, a finding which supports our results^41^. On the other hand, it is interesting that, while lack of function of CRB1 in human retina causes retinal dystrophies, this same mutation does not cause any apparent brain dysfunction or manifest functional alteration^17,29–32^. Our results showing an increase in CRB2 protein expression in the CRB1^rd8^ mouse brain, although exclusively in the cortex, and the fact that the levels of other proteins that cooperate in these functions are not considerably modified, may somehow explain the lack of an obvious brain phenotype. Further and more detailed studies regarding the collaborative interaction of these proteins in other areas of the brain are needed to confirm this hypothesis.

In conclusion, the novelties of our study are that it demonstrates the presence, distribution and localization of the CRB2 protein in mouse brain and also suggests that the increase in the levels of CRB2 in the CRB1^rd8^ mutant could respond to a possible compensatory mechanism that may counteract defects in the functioning of CRB1 in the mutant mouse model. Moreover, this is the first study providing proof that the CRB2 protein is only expressed in neurons in the brain, where it is localized in some neuronal presynaptic terminals, and in exocytic and endocytic vesicles.

## Materials and Methods

### Animals

All procedures used in this work were in accordance with the guidelines of the European Communities Council Directive 2010/63/UE and Spanish legislation RD 53/2013 for the use and care of animals. All the details of the study were approved by the Bioethics Committee of the University of Salamanca. For this study, 14 adult (post-natal or P) 180 to 200-day-old wild-type C57BL/6J and mutant C57BL/6J CRB1^rd8^ mice were used to carry out the immunofluorescence analysis; 15 adult (P180–P200) mice of both genotypes for the Western Blot analysis (WB) and 6 adult (P120) mice for the reconstruction of the cortex and quantification of the immunofluorescence labeling using the ImageJ software.

### Western blot analysis

Mice were euthanized with carbon dioxide. Portions of the Cortex (Cx), Hippocampus (Hc) and Cerebellum (Cb) areas were lysed in RIPA buffer (150 mM sodium chloride, 1% Triton X-100, 0,5% sodium deoxycholate, 0,1% sodium dodecyl sulphate, 50 mM Tris, pH 8.0) containing protease inhibitor cocktail (1:1000 Sigma-Aldrich®). Each piece of tissue was homogenized in different tissue grinders, maintained under constant shaking for 2 h at 4 °C and centrifuged at 15.000 g for 20 min at 4 °C. The supernatants were collected, the amount of protein was measured using the Bradford’s assay, mixed with 20% Bio-Rad Protein Assay Dye Reagent Concentrates (Bio-Rad Laboratories™) and absorbance at 595 nm was measured with a LT40000 Microplate reader (Labtech®). The proteins were then dissolved in sample buffer (2% sodium dodecyl sulphate SDS, 10% glycerol, 700 mM β-mercaptoethanol, 62.5 mM Tris-Hcl pH 6.8, 0.05% bromophenol blue) and loaded onto a SDS-polyacrylamide gel under reducing conditions. The proteins were transferred to nitrocellulose membranes, blocked for 1 h at room temperature (RT) in a solution with 2% bovine serum albumin (BSA) in Tris-buffered saline-Tween (0.1%) (TBST) and immunolabeled overnight at 4 °C with the primary antibodies of CRB2 (custom-made^20^, 2,25 µg/ml), β-actin (Sigma-Aldrich®, 1:5000), Par3 (Millipore, 1:250), β-catenin (Santa-Cruz Biotechnology®, 1:100) and Occludin (Invitrogen™, 1:200) in a solution of 2% BSA in TBST. After several washes in TBST, the membranes were incubated with 1:10000 anti-rabbit IgG, 1:10000 anti-goat IgG, 1:10000 anti-mouse IgG (Jackson ImmunoResearch™) or 1:10000 protein-A and 1:10000 protein-G (Life Technologies™) conjugated with horseradish peroxidase in 2% BSA and 2% nonfat dried milk in TBST for 60 min. At RT, the membranes were washed with TBST and developed with Pierce™ ECL Plus Western Blotting Substrate (Thermo Scientific). As negative controls, the nitrocellulose membrane was incubated without the primary or secondary antibodies and without both.

### Immunofluorescence

The animals were anaesthetized with chloral hydrate 5% (p/v) in NaCl 0.9% (p/v), and perfused transcardially with a solution containing 4% paraformaldehyde in 0.1 M phosphate buffer at pH 7.4 (PB) and the brains were dissected out and post-fixed by immersion for 2 h at RT in the same fixative. Later, brain tissue was cryoprotected with a 30% sucrose solution in 0.1 M PB at pH 7.4, embedded in OCT (Tissue-Tek® O.C.T. ™) and 30 µm sections were obtained using a freezing-sliding microtome (Leica® frigomobil, Jung SM 2000 Leica®, Nusscoch Germany). Sections were washed in a PBS solution (0.1 M, pH 7.4) and then stored at 4 °C in a freezing solution (30% glycerol and 30% ethylene glycol in 0,1 M PB at pH 7.4). For immunolabeling, sections were rinsed in PBS with Triton Tx-100 at 0.2% (Sigma-Aldrich®) (PBS-Tx) and blocked for 2 h with 1% BSA and 5% normal serum in PBS-Tx. Section were then incubated overnight at 4 °C with 1% BSA, 2% normal serum and the primary antibodies for CRB2 (custom-made^20^, 2,25 µg/ml), Pals1 (Abnova, 1:100), Synaptophysin (Sigma-Aldrich®, 1:5000), PSD95 (Affinity Bioreagents™, 1:500), V-Glut1 (Synaptic Systems, 1:500), VPS35 (Santa Cruz®, 1:50), Rab8A (Abcam®, 1:100), Calbindin (Swant®, 1:1000), Tuj1 (Biolegend®, 1:500), Iba1 (Santa-Cruz Biotechnology®, 1:250), Olig1 (Everest Biotech, 1:100), MAP2 (Sigma®, 1:1000) and Sinapsin 1a/b (Santa-Cruz Biotechnology®, 1:200). Following on, the sections were washed with PBS and PBS-Tx and incubated for 90 min at RT with 1:250 Cy3 and 1:250 Cy2 (Sigma-Aldrich® Jackson ImmunoResearch^™^) or 1:750 Alexa fluor 488, 1:750 Alexa fluor 555 and 1:750 Alexa fluor 633 (Thermo Fisher) fluorescent secondary antibodies and DAPI (1:10000). The sections were cover-mounted using Prolong® Gold antifading reagent (Life Technologies™). Also, negative controls were prepared by excluding the primary or secondary antibodies in the incubation steps.

### Immunohistochemistry

Selected sections were labelled using the Avidin-Biotin Complex (ABC) method. After cryoprotection, the sections were washed in a PBS solution (0.1 M, pH 7.4) and then rinsed in a solution with PBS, methanol and 30% H_2_O_2_ to remove endogenous peroxidase activity within the tissue. After rinsing with PBS-Tx (0.4 M, pH 8), the sections were incubated with the CRB2 antibody (custom-made^20^, 2,25 µg/ml at 4 °C for 72 h. Next, the sections were washed in PBS-Tx (0.4 M, pH 8) and incubated for 2 h with the secondary anti rabbit Ig-G biotinylated antibody (Jackson Immunoresearch Laboratories) conjugated to horseradish peroxidase in PBS-Tx. The sections were incubated for 3 h in a solution with PBS-Tx (0,4 M, pH 8) and the avidin-biotin peroxidase kit (ABC complex, Vector) and labeling was developed using 3,3′-diaminobenzidine tetrahydrochloride (DAB 0,2%) as chromogen. The negative staining controls were prepared without the primary or secondary antibodies.

### Imaging

Images were obtained using different types of microscopes: an epi-fluorescence microscope, Olympus® Provis AX70, coupled to a digital DP Olympus® camera; a laser scanning spectral confocal microscope, Leica® TCS SP2, with the pinhole set at 1.2 Airy Units and 40x and 63x immersion oil objectives (488 nm and 543 nm were used to excite Cy2 or Alexa 488 and Cy3 or Alexa 555 fluorochromes, respectively, and 633 nm was used to excite the TOPRO3 fluorochrome); and an inverted Zeiss Axio Observer Z1 microscope (Carl Zeiss Microscopy, UC, USA) coupled to an Axio Cam MRm Zeiss® camera for live-cell imaging. The images were captured and processed using Leica Confocal Software (DP controller software Leica®) and ZEN 2011 imagen software (Carl Zeiss Microscopy). The brightness and contrast of all original images were further processed and adjusted using Adobe Photoshop CS6.

### Statistical analysis

All statistical analysis were performed using Microsoft Excel (Microsoft® Office 2013) and SPSS (IBM, Armonk, NY) software. The Kolmogorov Smirnov test was used to assess the normality of sample distribution. Two experimental groups were compared with the Student’s T test for parametric data, and Mann Whitney U test for not parametric data. More than three groups were analyzed with one way-factorial ANOVA and the Bonferroni’s post-hoc test for parametric data, and the Kruskal Wallis test for not parametric data. Values were expressed as mean ± standard error for the mean (SEM). Values of *P < 0.05 were considered significant and **P < 0.01 highly significant. Values non-significant were indicated by ns.

## Electronic supplementary material

Supplementary figure
