# Supplementary material for: Expression and localization of the polarity protein CRB2 in adult mouse brain: a comparison with the CRB1rd8 mutant mouse model
Source: Sci Rep. 2018 Aug 3;8:11652. doi: 10.1038/s41598-018-30210-5 (PMC6076319; doi:10.1038/s41598-018-30210-5)
Supplement: Supplementary file 1 — Supplementary figure [file 41598_2018_30210_MOESM1_ESM.pdf]

Expression and localization of the polarity protein CRB2 in adult mouse brain: a comparison with the CRB1rd8 mutant mouse model

Jorge F. Dolón, Antonio E. Paniagua, Vicente Valle, Alicia Segurado, Rosario Arévalo, Almudena Velasco and Concepción Lillo*

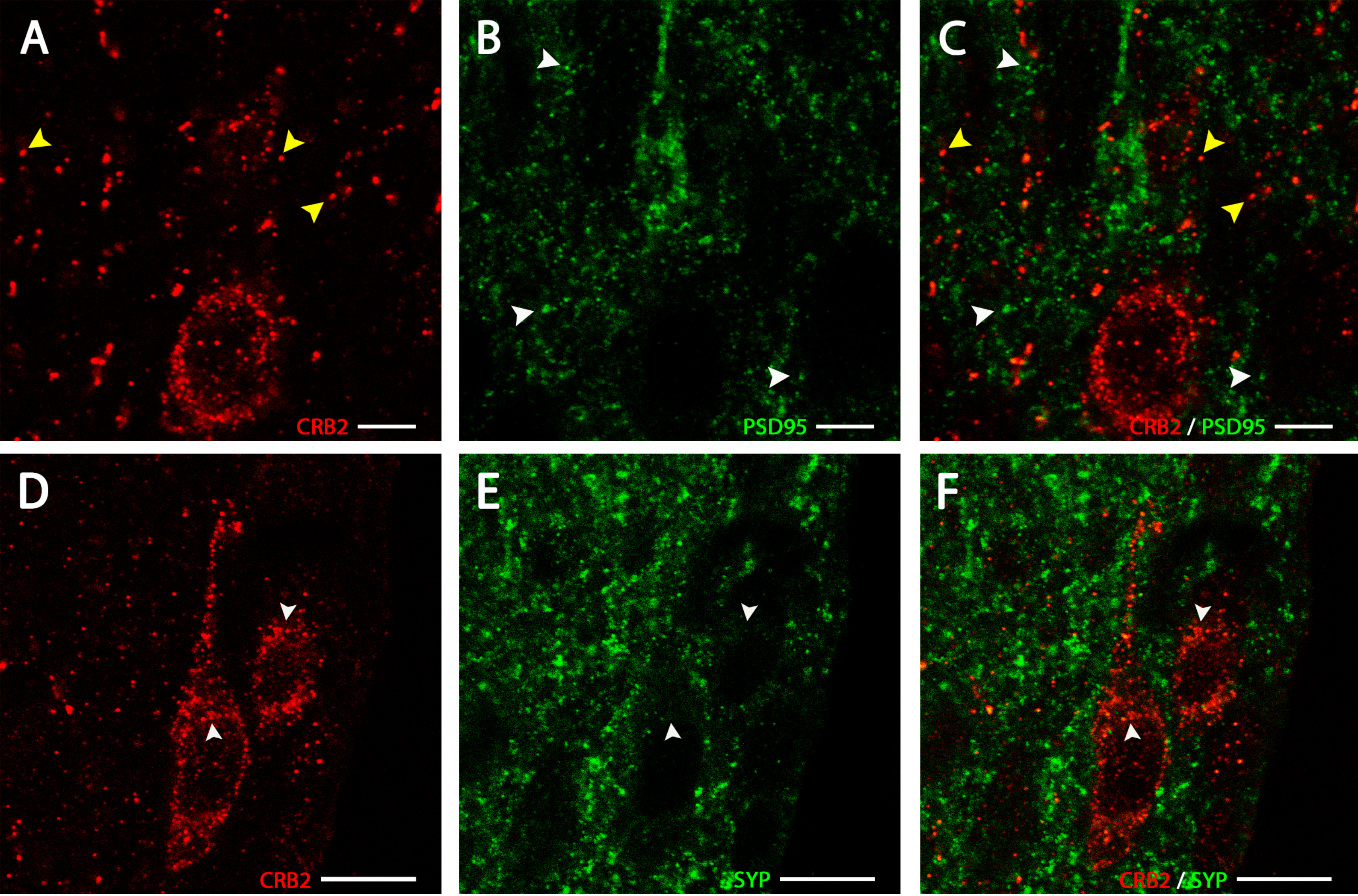

Supplementary figure 1

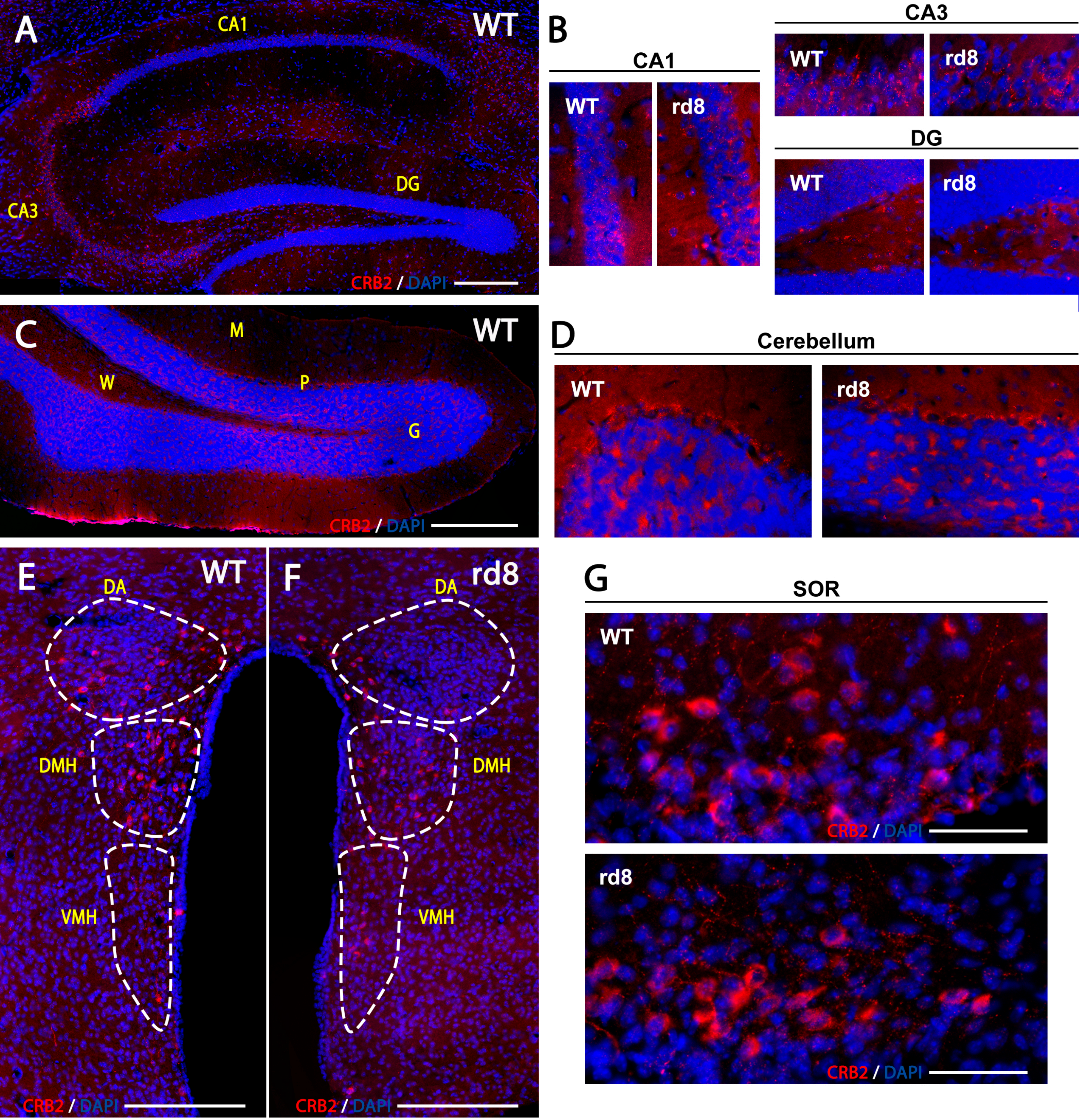

Supplementary figure 2

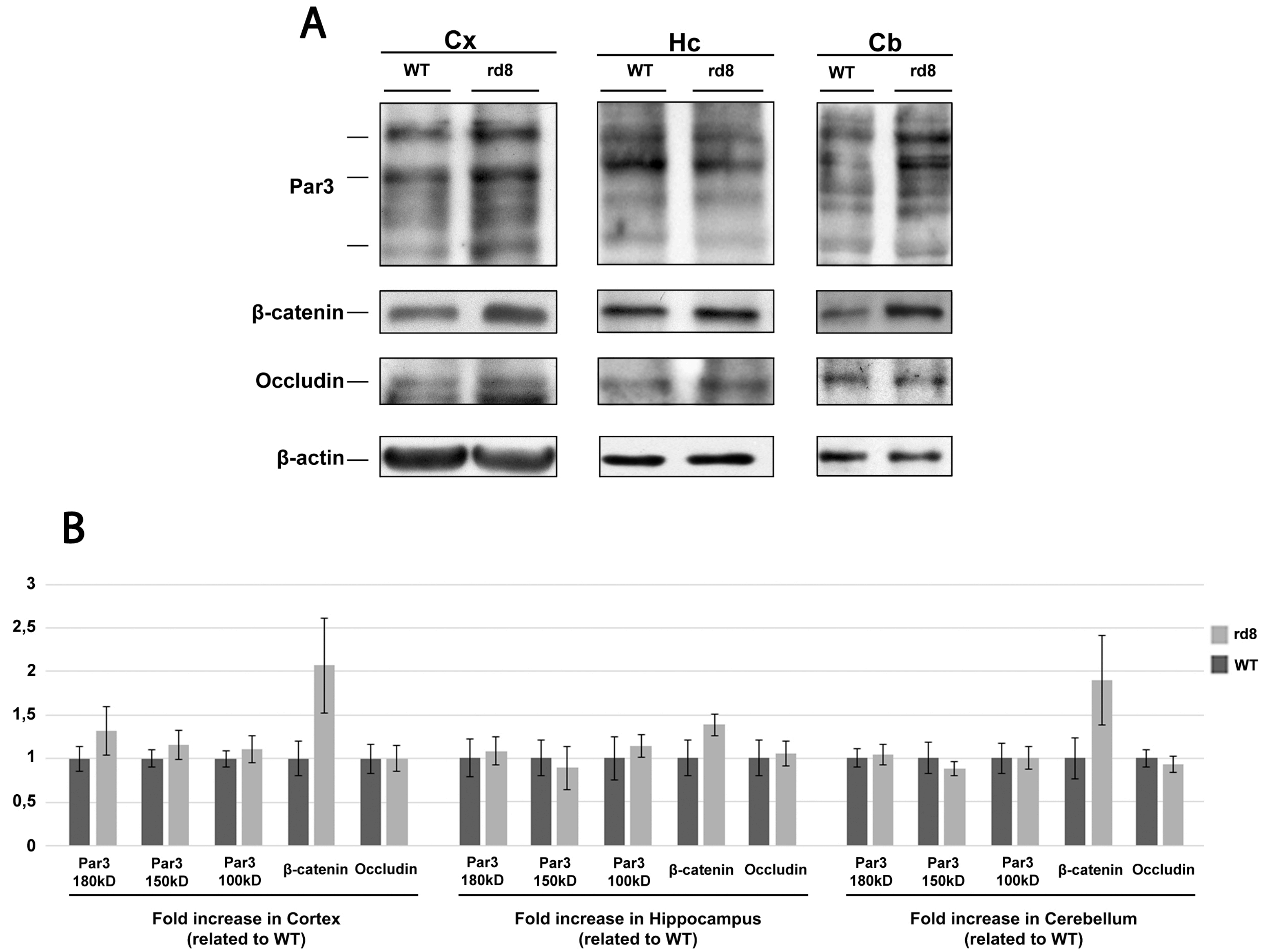

Supplementary figure 3

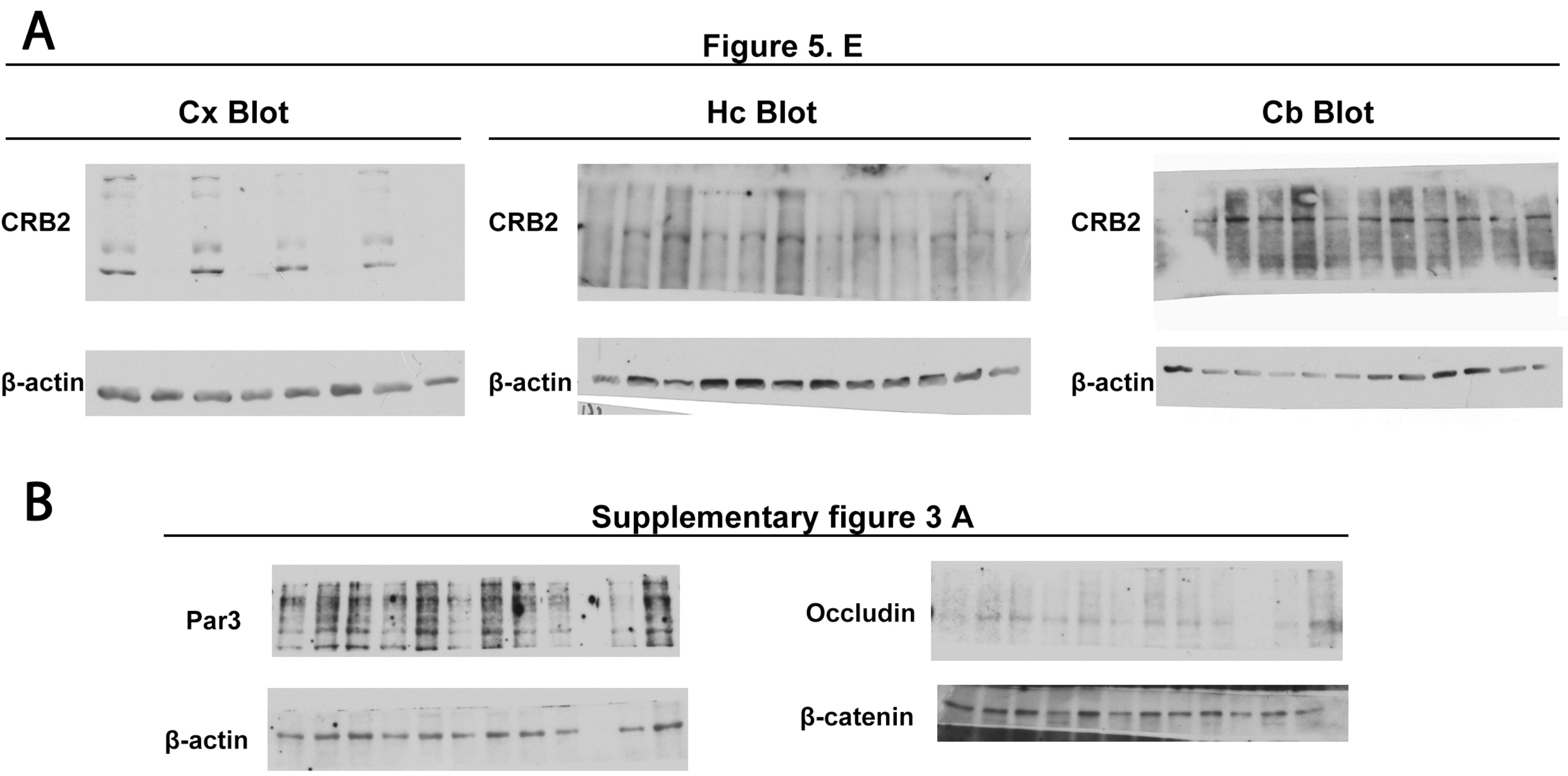

Supplementary figure 4

Supplementary figure 1. CRB2 and synaptic proteins in the cell bodies of neurons. Double immunofluorescence analysis of CRB2 (**A, C, D, F**) and PSD95 (**B, C**) and SYP (**E, F**) in coronal sections of adult mouse PVN. In the cell bodies, CRB2 (yellow arrowheads) and PSD95 (white arrowheads) do not colocalize (**A-C**) and CRB2/SYP colocalization is frequent in the profiles scattered throughout the sections (Fig.4, D-F), but not in the soma of neurons (**D-F**). Scale bars: 5 µm (A-C), 10 µm (D-F).

Supplementary figure 2. Comparative CRB2 distribution in brain. A: Immunofluorescence of CRB2 distribution in a hippocampal sagittal section of WT adult mouse brain. **B:** Comparative CRB2 labeling from similar selected regions of the CA1, CA3 y DG hippocampal areas from WT and CRB1rd8 mice brain. **C:** Immunofluorescence of CRB2 distribution in a cerebellum sagittal section of WT adult mouse brain. **D:** Comparative CRB2 labeling of a selected region of the Purkinje cells' layer from WT and CRB1rd8 mice brain. **E-F:** Comparison of the CRB2 labeling distribution in the different hypothalamic nuclei and areas of WT (**E**) and CRB1rd8 (**F**) mice brain. **G:** Comparative CRB2 labeling in the SOR area of WT and CRB1rd8 mice brain. DG (dentate gyrus), M (molecular layer), P (Purkinje cells' layer), G (granular layer), W (white matter), DA (dorsal area), DMH (Dorsomedial Hypothalamic Nucleus), Ventromedial Hypothalamic Nucleus (VMH), SOR (Retrochiasmatic Supraoptic Nucleus). DAPI (in blue): nuclear labeling. Scale bars: 200 µm (A-F), 50 µm (G).

Supplementary figure 3. Comparative expression of CRB2, Par3 and cell adhesion proteins in WT and CRB1rd8 mice cortex, hypothalamus and cerebellum. A: WB analysis showing the comparative expression of Par3 (long form 180 kD, medium form 150 kD and short form 100 kD) and cell adhesion proteins (Occludin 65 kD and β-Catenin 92 kD) in WT and CRB1rd8 mice cortex, hypothalamus and cerebellum. **B:** Graph showing the comparative quantification of the proteins analyzed in **A** in WT and CRB1rd8 mice cortex, hypothalamus and cerebellum, respectively. Data are presented as mean ± s.e.m. β-actin was used as the loading control. Data are presented as mean ± s.e.m. Statistical information: The Kolmogorov Smirnov test was used to assess the normality of sample distribution. In **B**, in all cases, two experimental groups were compared with the Mann Whitney U test (n = 15). Blots were cropped from the same gel and quantitative comparisons were performed between samples of the same blot. Full-length blots are presented in Supplementary Figure 4.

S**upplementary figure 4. Originals blot of western blot experiments. A:** Some of the original blots of the WB analysis shown in Figure 5E showing the analysis of CRB2 expression in Cx, Hc and Cb. **B:** Some of the original blots of the WB analysis shown in Supplementary figure 3A showing the comparative expression of proteins such as Par3, Occludin and β-Catenin in WT and CRB1rd8 mice brain.
